# Acute Rheumatoid Arthritis

**Published:** 1908-10-31

**Authors:** 


### SPECIAL ARTICLE.

### ACUTE RHEUMATOID ARTHRITIS.

The varieties of multiple acute, or subacute, joint lesions are still the subject of great controversy; for although acute rheumatic arthritis, gonococcal arthritis, pyæmic arthritis, post-scarlatinal arthritis, acute suppurative arthritis, acute gouty arthritis, chronic osteo-arthritis, and Charcot's arthritis of tabes dorsalis and syringo-myelia, have been differentiated with fair clearness from the main mass of cases, there still remain many which have not been so differentiated. The name "rheumatoid arthritis" is often applied promiscuously to conditions which have very different clinical characters. One of the reasons for the difficulty in classification is probably the attempt to make pathological distinctions coincide with clinical groups; or rather perhaps to break up the clinical groups in such a way that they will coincide with the pathological and bacteriological causes of the joint lesions. The attempt is much to be lauded; but the classification which results may not be of real service in clinical medicine if the same organism may sometimes produce one variety of joint lesion and sometimes another; or if the same appearances as regards the joints may be produced sometimes by one organism, sometimes by another.

To take an analogy: the streptococcus longus may cause acute peritonitis, or otitis media, or erysipelas, or fungating endocarditis; but it would be the reverse of helpful to describe fungating endocarditis as essentially similar to acute peritonitis. On the other hand, acute peritonitis may be due to the streptococcus longus, the bacillus coli communis, the pneumococcus lanceolatus, the staphylococcus pyogenes aureus, and so forth; but although all these varieties have a different bacteriological basis, it would not be clinically helpful to insist too much upon their being essentially different diseases.

So with regard to arthritis. The staphylococcus may affect a few joints, causing suppuration in each, with fatal result; or it may affect many joints, causing painful swelling and subsequent partial or complete limitation of movement, but not leading to any pus formation. Bacteriologically, the two kinds of joint lesion are similar, or even identical, except in degree. Clinically the organism at work is of less significance than is the result in the affected joints. The suppurative lesion would be classed with other suppurative arthritides, whether due to the staphylococcus or to any other organism. Multiple non-suppurative staphylococcal synovitis would be classed, clinically, with other multiple non-suppurative synovitides, due to other organisms than the staphylococcus—the pneumococcus, the streptococcus, streptothrix infections, and so forth. It is probable, indeed, that the same clinical picture may be produced by various different organisms in different cases; and although from the point of view of treatment by vaccines and the like it becomes very necessary to know what organism is at work in each case, the clinical characters of the disease are better classified under the heading of the resultant lesions than under that of the organism which has produced them.

Even if this is not agreed to by all, or if it is not the ideal that should be aimed at in the future, it is the best ground for the classification of multiple subacute joint lesions at present. Thereby we are able to recognise at once one very typical variety which, for lack of better terms, is called either *acute rheumatoid arthritis* or *multiple septic synovitis*. The disease affects women much more commonly than men, and young women between the ages of 20 and 35 in particular. Its distinguishing mark is the spindle-shaped enlargement of all the first interphalangeal joints of the fingers. There is scarcely a joint in the body that may not be involved as well, but the condition of these first interphalangeal joints is what gives the diagnosis of this particular type of joint affection at once. The joint next most commonly affected is the wrist, which becomes bulged both dorsally and upon the palmar surface; the elbows and shoulders come next; all the other joints are more rarely affected, though some cases can move neither hand nor arm, foot nor leg, from involvement of every joint in both limbs. The main complaint is that of weakness in the hands, so that the patient, though willing to work, is quite unable to do so. The weakness is partly due to the unbending state of the affected joints, but also partly to actual loss of power in the muscles. It is persistent. Pain is the other main symptom; it is not constant, like the weakness, but varies from time to time. There are periods during which the least movement of the joints causes excruciating pain; and there are other times when the pains are absent, or only slight. The patients are nearly always thin, and there is a particular tendency to freckle-like pigmentation of the limbs and trunk.

The bacteriology of the lesion is almost unknown. Whenever there is an increase in the pain and swelling in the joints, or an extension to new joints, there is pyrexia. The latter is slight in degree—going up to 99.6° F. or 100° F. each night—but it may persist for many weeks at a stretch. During this time the joint trouble is in an active stage; as the synovitis subsides, so does the pyrexia, and an interval of weeks, months, or years may elapse without any further change. Sooner or later, however, the permanent deformity left by one attack is increased by further exacerbations.

Another point in favour of there being a microbial infection is the fact that in nearly all these cases, particularly during a pyrexial period, there is some enlargement of the lymphatic glands nearest to the affected joints. The epitrochlear glands are those most commonly to be felt, as might be expected from the fact that the most prominently affected joints are in the fingers; the axillary glands are palpable, hard, and sometimes tender, nearly as often. The glands in the groin are less frequently big; whilst the cervical groups are involved least commonly of all. If one recalls what happens in the precisely similar joint disease in children—Still's disease—one might expect that the spleen would be palpable also; this, however, is not the rule in acute rheumatoid arthritis of adults.

The $x$-rays show no alteration in the outlines of the bones, but only a rarefaction of their tissue. The lesion is essentially in the synovial membrane, the ligaments, and the periarticular tissues. It is on this account that the alternative name of multiple septic synovitis seems justified.

The treatment of acute rheumatoid arthritis is very tedious and disappointing; for do what one will, it is almost certain that exacerbations of the joint trouble will recur from time to time, and whatever care one takes each exacerbation leaves behind it some addition to the previous crippling of the joints.

The treatment is essentially different at the time when the patient is suffering from an exacerbation to what it is between the acute attacks. In the quiescent interval considerable therapeutic activity is advisable, whereas when the synovitis is itself active nothing drastic should be attempted, and the joints should be kept as still as possible until the active inflammatory changes have begun to subside again. If one may dogmatise upon the subject, one would say that in all cases any discoverable source of septic absorption should be attended to; that in acute stages rest in bed, with the least possible movement of the joints, is indicated, together with a diet containing an abundance of proteid; that between the acute exacerbations the joints will benefit much from radiant-heat baths, massage and active and passive movements, especially at certain spas; and that no drugs, vaccines, or sera have been found to exert any specific action upon the condition, although salicylates, iodides, cod-liver oil, and arsenic seem to be of greater general service in the disease than any other medicines.

We do not yet know the nature of the organism that produces acute rheumatoid arthritis; indeed, we do not know whether the disease is specific or whether it may not have several different bacterial causes. Nevertheless, if a patient is also suffering from any pyogenic process such as pyorrhœa alveolaris, it is clearly wise to cure the latter, if possible.

The general rule for the treatment of osteoarthritis is that continued rest is bad; in acute rheumatoid arthritis, on the other hand, there are stages when complete rest is strongly indicated. These stages are those during which there is pyrexia. The patient's temperature is seldom high, but if it is taken night and morning it continues above the normal line for days or weeks at a time. This pyrexia is associated with an increase in the painfulness and swelling of the already affected joints, and possibly a spread of the process to joints not hitherto involved; the neighbouring peripheral lymphatic glands become more enlarged at the same time, and everything points to there being an increase in the activities of the microbes in the joints. At such times complete rest is indicated, preferably in bed, the joints being protected from exposure to variations in temperature or to injury by being gently swathed in warm cotton wool. Of all the points which indicate an exacerbation in the synovitis the temperature chart is the most helpful, whereas the pains in the joints are very misleading, for they may be almost as severe in the quiescent intervals as during the acute attacks. Whatever the pains, it is only when there is pyrexia that the joints must be kept at rest.

It has been urged that a diet which contains a relatively large amount of proteid is the best for patients suffering from rheumatoid arthritis. There are some observers who believe so thoroughly in this that they prescribe meat or fish or eggs to every meal in the day, and in quantities sufficient to reduce carbohydrates and fats to a minimum. So extreme a course is probably unwise; it is sufficient if the general principle that a relatively generous proteid dietary is acted upon rationally in each case, the patient being advised to partake freely of meats, fish, eggs, and other proteid foods.

There is no drug which exerts any specific action upon the joints in acute rheumatoid arthritis. Sodium salicylate and its allies sometimes seem to do good, but the relief to the pain and swelling comes very slowly, and salicylates exert practically no influence upon the temperature in the pyrexial stages of the malady. A great many other drugs have been given in different cases; sometimes the administration has seemed to give some relief, at other times none. In the future it seems probable that successful treatment upon the lines of vaccine may be devised. Iodide of potassium, iodide of iron, perchloride of mercury, sulphocarbolate of sodium, $\beta$-naphthol, glycerine of carbolic acid, vinum colchici, and a host of other drugs have been used. Whatever drug is tried, its use should be persevered with for some weeks before it is given up as of no value in the case. The general opinion seems to be that arsenic is the most valuable of all, beginning with doses of 4 minims of Fowler's solution in an ounce of chloroform water three times a day after meals, and increasing the dose by 1 minim every week or ten days until the patient is taking as much as 10 or 12 minims to each dose. The arsenic should be given chiefly in the acuter phases, and continued as long as there is pyrexia.

Radiant-heat baths are indicated, not during an exacerbation, but during the quiescent intervals, with a view to diminishing the periarticular thickening and making the joints less stiff. The simplest method of giving a radiant-heat bath is by means of powerful electric lights suspended within a cradle, beneath which the part to be treated is inserted, the whole being surrounded with blankets and mackintosh sheeting to form a closed space containing the electric lamps and the joints that are to be dealt with. Thirty-two candle-power lamps are needed as a rule, and four of them are sufficient to produce by their radiant heat a temperature of 150° C. to 200° C. within the cradle chamber. The joints are exposed to this heat for a quarter of an hour, twenty minutes, or half an hour, as the case may be, once a day. After each application the affected parts become very much more lissom, and passive movements can be made through a range that was quite impossible before the heat bath. Unfortunately the stiffness comes on again as the joint cools, notwithstanding massage and passive movements. The process has to be repeated daily for weeks before marked permanent improvement is to be expected.

There is no particular virtue in the electric lamps beyond the fact that they are a convenient source of radiant heat. Nearly as much benefit can be obtained by hot-sand baths as described in a former issue; the essential thing seems to be the hot, dry heat.

